# Incidence and outcomes of multidrug-resistant gram-negative bacteria infections in intensive care unit from Nepal- a prospective cohort study

**DOI:** 10.1186/s13756-018-0404-3

**Published:** 2018-09-26

**Authors:** Shraddha Siwakoti, Asish Subedi, Abhilasha Sharma, Ratna Baral, Narayan Raj Bhattarai, Basudha Khanal

**Affiliations:** 1 Department of Microbiology, B. P. Koirala Institute of Health Sciences, Dharan, 56700 Nepal; 2 Department of Anaesthesiology and Critical care, B. P. Koirala Institute of Health Sciences, Dharan, Nepal

**Keywords:** ICU, Multidrug-resistant gram-negative bacteria, Healthcare-associated infection, Incidence, Outcome

## Abstract

**Background:**

Infections caused by multi-drug resistant gram-negative bacterial infections are the principle threats to the critically ill patients of intensive care units. Increasing reports of these infections from the Nepalese intensive care unit underline the clinical importance of these pathogens. However, the impact of these infections on the patient’s clinical outcome has not yet been clearly evaluated. The objective of our study was to determine the incidence and associated clinical outcome of multi-drug resistant gram-negative bacterial infections in intensive care unit from a tertiary care center of Nepal.

**Methods:**

A prospective cohort study was conducted among adult patients admitted in intensive care unit of B. P Koirala Institute of Health Sciences from July to December 2017. Patients infected with multi-drug resistant gram-negative bacteria, non-multi-drug resistant gram-negative bacteria and those without infection were included. Identification of gram-negative bacteria and their antibiotic susceptibility pattern was performed with standard microbiological methods. Demographic, clinical profiles and outcomes (in-hospital-mortality, intensive care unit and hospital length of stay) were documented.

**Results:**

The incidence rate of multi-drug resistant gram-negative bacteria infections was 47 per 100 admitted patients (64/137) with 128 episodes. *Acinetobacter species* (41%, 52/128) was the commonest followed by *Klebsiella pneumoniae* (28%, 36/128) and *Pseudomonas spp* (21%, 27/128). Patients with multi-drug resistant gram-negative bacteria in comparison to non-multi-drug resistant gram-negative bacteria had high healthcare-associated infections (95%, 61/64 versus 20%, 2/10; *p* = < 0.001). In-hospital-mortality was 38% (24/64), 20% (2/10) and 10% (4/41) in multi-drug resistant, non-multi-drug resistant and uninfected group respectively (*p* = 0.007). After adjustment for independent risk factors, compared to uninfected patients, the odds ratio (CI) for in-hospital-mortality in multi-drug resistant and non-multi-drug resistant group was (4.7[1.4–15.5], *p* = 0.01) and 2.60 [0.38–17.8], *p* = 0.32) respectively. Multi-drug resistant patients also had longer intensive care unit and hospital stay, however, it was statistically insignificant.

**Conclusion:**

The incidence of multi-drug resistant gram-negative bacterial infections was remarkably high in our intensive care unit and showed a significant association with healthcare-associated infections and in-hospital-mortality.

## Background

The prevalence of infection is high among patients admitted to intensive care units (ICUs) and it is a major cause of mortality [1, 2]. The extended prevalence of infection in intensive care study reported infection in 51% of patients with gram-negative bacteria (GNB) isolation from 62% of infectious episodes [2]. As a disastrous effect of infection, antimicrobial resistance is an increasing concern in ICUs worldwide [3]. The global scenario shows that gram-positive infections are common in the developed countries ICUs [4]. However, multidrug-resistant gram-negative bacteria (MDR-GNB) infections dominate in the Asia-Pacific region [4, 5] including Nepal [6, 7]. Among MDR-GNB, extended-spectrum beta-lactamases (ESBL) organisms, carbapenemase producing enterobacteriaceae, carbapenem-resistant *Acinetobacter species*, multidrug-resistant *Pseudomonas aeruginosa* are the major culprits. Unfortunately, new antibacterial agents have not been developed in pace with the growth of multidrug-resistant (MDR) organisms [8]. There are now a rising number of reports globally [9] and also from Nepal [6, 7] of MDR-GNB infections in ICUs for which the treatment options are limited. The impact of the MDR-GNB infections can be determined from analyzing clinical outcomes, in-hospital-mortality and the length of ICU or hospital stay [10]. The association of MDR-GNB with a prolonged hospital length of stay (LOS) and mortality remains controversial. Several studies [10, 11] have reported the direct association whereas, others [12, 13] have shown that MDR-GNB infections are not associated with increased hospital LOS and mortality. Previous studies from Nepal have reported a high incidence of MDR-GNB infections from ICU [6, 7], but the impact of these infections on clinical outcome has not been evaluated. Therefore, the objective of our study was to determine the incidence of MDR-GNB infections in the critically ill patients from adult ICU, as well as the clinical outcomes with regard to in-hospital-mortality, ICU and hospital LOS.

## Methods

### Study design

This prospective cohort study was conducted in seven bedded general adult ICU under the care of the department of Anesthesiology and Critical care unit, B.P Koirala Institute of Health Sciences (BPKIHS), Nepal.

### Study population

All consecutive adult patients admitted to the medical ICU from July to December 2017 were eligible for the study. Patients infected with MDR-GNB, non-MDR-GNB and those without infection were included.

### Microbiological procedures

Pathogenic bacteria isolated from the clinical specimens from the ICU were further characterized by conventional biochemical tests to identify the specific GNB by using standard microbiologic methods [14]. Antibiotic susceptibility test of GNB strains was done by the Kirby Bauer disc diffusion method on Mueller Hinton agar (MHA) as per the Clinical Laboratory Standard Institute (CLSI) guidelines [15]. Antibiotics of following concentrations were used: ampicillin (10 μg), amikacin (30 μg), gentamycin (10 μg), tobramycin(10 μg), ciprofloxacin (5 μg), levofloxacin (5 μg), chloramphenicol (30 μg), co-trimoxazole (25 μg), ceftazidime (30 μg), cefotaxime (30 μg), cefepime (30 μg), piperacillin (100 μg), carbenicillin (100 μg.), piperacillin-tazobactam (100/10 μg), imipenem (10 μg), tigecycline (30 μg), polymyxin B (300unit), and colistin sulphate (10 μg) from HiMedia Laboratories, India. Disk zone diameters were interpreted according to the CLSI 2017 recommendations. Quality control for culture plates and antibiotic susceptibility was performed using *Escherichia coli* ATCC 25922 and *Pseudomonas aeruginosa* ATCC 27853. All the strains were subjected to various phenotypic methods for the screening and confirmation of the beta lactamases. Strains showing decreased sensitivity to ceftazidime/ cefotaxime were considered as screen positive for ESBL production and were subjected to the following confirmatory phenotypic tests as per the CLSI guidelines [15].

• ESBL- A difference in the zone size of 5 mm between ceftazidime and ceftazidime+ clavulanic acid and cefotaxime and cefotaxime+clavulanic acid discs was considered as confirmed ESBL producer [15].

• Carbapenemase- The screen positive for carbapenemase production was considered for strains showing resistance to carbapenems. A positive modified hodge test (MHT) with appearance of clover leaf at the streaking line was considered as carbapenemase producer as per the CLSI guidelines [15]. A difference in the zone size of 7 mm between Imipenem and Imienem+ EDTA disc in the EDTA disk synergy test was considered as MBL producer [16].

### Definitions

Infection-An episode of infection was defined as the isolation of GNB in the presence of compatible signs or symptoms. Healthcare-associated infections (HCAI) and those infections present on admission were included.

Infection occurring > 48 h after admission to the hospital was defined as HCAI.

MDR was defined as non-susceptibility to at least one agent in three or more antimicrobial categories [17].

Diagnostic criteria recommended by CDC was implemented to classify different infections. Pneumonia was considered if purulent tracheobronchial secretion or new pathogenic bacteria isolated from sputum or tracheal aspirate culture with ≥10 [4] colony forming unit/ml and at least two of the following criteria were met: fever (> 38°C); leukocytes > 12,000 or < 4000 cells/ml; new or progressive pulmonary infiltrates on chest X-rays; new onset or worsening cough or dyspnea or tachypnea; or worsening gas exchange.

An episode of blood stream infection (BSI) was defined as one positive blood culture with a recognized pathogen or two positive cultures with same organism drawn on separate occasions with one of the following signs and symptoms: (fever(> 38°C), chills and rigor and hypotension.

An episode of urinary tract infection (UTI) was defined as a positive urine culture of ≥10 [5] colony forming units/ml and with no more than two species of microorganisms, and at least one of following signs or symptoms: fever (> 38°C); dysuria; suprapubic tenderness; costovertebral angle pain or tenderness with no other recognized cause.

An episode of surgical site infection (SSI) was defined as infection which occurred within 30 days after the operation involving skin, subcutaneous tissue or deep soft tissue of the incision and at least one of the following: purulent drainage with or without laboratory confirmation; organisms isolated from an aseptically obtained culture of fluid or tissue; or one of the signs or symptoms of infection: pain or tenderness, localised swelling, redness, or heat.

Based on the presence or absence of infection, patients were categorized into three groups: Uninfected patients- Patients without infection; Non-MDR-GNB patients-Infections attributed to susceptible GNB and MDR-GNB patients- Infections attributed to MDR-GNB.

Patients were included more than once in the analysis for separate episodes of infection.

In cases of polymicrobial infections, the episode was defined as an MDR-GNB case if 1 of the isolates was an MDR-GNB strain.

Previous antibiotic therapy was defined as antibiotic used within 30 days prior to positive culture for GNB.

Empiric antibiotic therapy was considered inappropriate if it did not include at least one antibiotic active against the GNB in vitro. Empirical antibiotic treatment protocols were same for all the groups and the antibiotic was changed after the culture and sensitivity report.

### Data collection

Patient demographic characteristics, underlying conditions and reason for hospital admission were recorded in the participant record form at the time of admission. Patient were routinely followed up again each morning and data on clinical or laboratory parameters were collected, including previous antibiotic therapy, clinical manifestations, HCAI, pathogens and antibiotic resistance. The baseline severity of illness were assessed with acute physiology chronic health evaluation II (APACHE II) score [18] and Charlson comorbidity index (CCI) score [19]. Further, the data were collected regarding clinical outcomes that included the ICU stay, hospital stay, discharge and in-hospital-mortality.

### Statistical analysis

Data were entered in the MS Excel 2007 and analyzed with STATA version 14 (stata corporation, college station, Tx, USA). Normal distribution of data was tested using histogram, skewness-kurtosis, and shapiro–wilk test. We used kruskal–wallis test for non-parametric data to compare between three groups. Categorical data were analyzed using the chi-square test or fisher’s exact test as appropriate. Univariate and multivariate logistic regression analysis was used to compare in-hospital-mortality between the groups. Data are reported as median (IQR), number (percentage), odds ratio (95% confidence interval). Values of *p* < 0.05 was considered statistically significant.

## Results

**Fig. 1:**
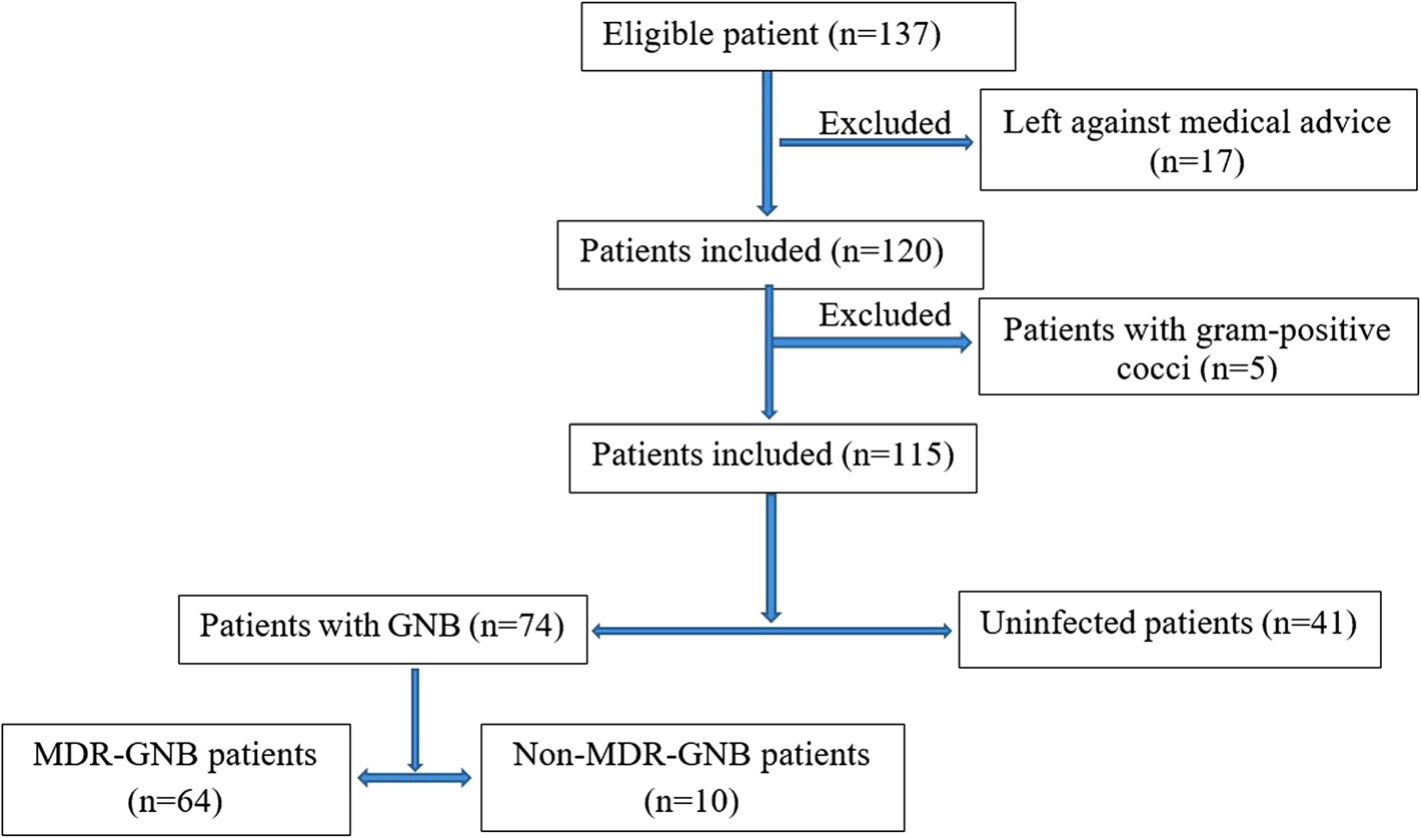
Flow diagram of the study population

A total of 137 patients were admitted to the ICU during the 6 months study period. There were128 episodes of MDR-GNB infections in 64 patients with an incidence rate of 47 per 100 ICU admissions. There were 41 uninfected and 10 infected cases with 19 episodes of non-MDR-GNB infections (Fig. 1).

Among the GNB infection episodes, incidences of MDR for each of the bacterial strains were reported as 100% (4/4) for *Enterobacter spp*, 100% (2/2) for *Citrobacter spp*, 93% (52/56) for *Acinetobacter spp*, 86% (36/42) for *Klebsiella pneumoniae,* 84% (27/32) for *Pseudomonas spp* and 64%(7/11) for *Escherichia coli*. Polymicrobial infection was present in 28% (18/64) MDR-GNB patients and 10% (1/10) in non-MDR-GNB patients. The detailed results of GNB pattern in the non-MDR-GNB and MDR-GNB group are presented in Table 1.

**Table 1:** Gram-negative bacilli (GNB) infections from ICU (*n* = 147)

Gram negative bacilli isolates	Total GNB	Non-MDR-GNB	MDR-GNB			
			Resistance mechanisms			Total MDR
			ESBL	CP(MBL)	Other	
*Acinetobacter spp*	56	4(21%)	6	34	12	52(41%)
*Pseudomonas spp*	32	5(26%)	3	19	5	27(21%)
*Klebsiella pneumoniae*	42	6(32%)	12	19	5	36(28%)
*Escherichia coli*	11	4(21%)	6	1	–	7(5.5%)
*Enterobacter spp*	4	–	2	2	–	4(3%)
*Citrobacter spp*	2	–	2		–	2(1.5%)
Total	147	19(100%)	31(24%)	75(59%)	22(17%)	128(100%)

In the MDR group, bacteria were most frequently isolated from the lower respiratory tract infection (LRTI) (72%, 92/128) followed by BSI (14%, 18/128), UTI and SSI each with (3%, 4/128). Whereas, in the non-MDR group, BSI (53%, 10/19) was the commonest followed by LRTI (42%, 8/19) and UTI (5%, 1/19). MDR-GNB showed variable degree of resistance to different classes of antibiotics as shown in Table 2.

**Table 2:** Antibiotic sensitivity of multidrug-resistant gram negative bacilli (*n* = 128)

Antimicrobial agents	Resistance (%) among bacterial isolates					
	*Acinetobacter spp* (*n* = 52)	*Pseudomonas spp* (*n* = 27)	*Klebsiella pneumoniae* (*n* = 36)	*Escherichiacoli* (*n* = 7)	*Enterobacter spp* (*n* = 4)	*Citrobacter spp* (*n* = 2)
Levofloxacin	85	88	73	57	100	50
Ciprofloxacin	92	90	82	86	100	100
Amikacin	93	89	76	71	100	50
Gentamycin	93	89	79	71	100	50
Tobramycin	–	87	–	–	–	–
Chloramphenicol	–	–	73	57	100	0
Cotrimoxazole	90	–	73	71	100	50
Ampicillin	–	–	–	100	100	100
Piperacillin	93	90	79		100	100
Piperacillin- Tazobactam	86	82	73	100	100	100
Amoxicillin-clavulanate	–	–	76	100	100	100
Ceftazidime	93	92	92	100	100	100
Cefotaxime	93	92	92	100	100	100
Cefepime	87	90	86	100	100	100
Imipenem	81	82	69	14	100	0
Tigecycline	58	63	57	14	67	0
Polymixin B	0	0	0	0	0	0
Colistin Sulphate	0	0	0	0	0	0

Demographic and clinical characteristics are provided in Table 3.

**Table 3:** Baseline and Clinical characteristics of patients

Variables	Uninfected patients; *n* = 41	Patients with MDR-GNB; *n* = 64	Patients with non-MDR-GNB; *n* = 10	*p*-value
Age (years*)*	43.5(28–56)	53(27–65)	55(40–60)	0.27
Age categories				
< 65	25(61%)	49(76%)	8(80%)	0.18
> 65	16(39%)	15(23%)	2(20%)	
Sex(M/F)	19/23	28/35	4/6	0.95
Reason for admission				
Cardiovascular	9(22%)	16(25%)	6 (60%)	0.89
Respiratory	27(66%)	44 (69%)	3(30%)	
Digestive/Liver	1(2%)	2(3%)	0 (0%)	
Renal	1(2%)	2(3%)	1 (10%)	
Neurological	3(7%)	0 (0%)	0(0%)	
Medical/Surgical admission	35/6	50/14	7/3	0.47
CCI Score	0(0–2)	1(0–3)	1(0–1)	0.77
APACHE Score				
At 24 h	13.5(11–16)	16(12–21)	13(12–15)	0.15
At 48 h	13(11–15)	17(12–20)	13(12–14)	0.08
Duration of ventilation	0(0–7)	10(6–16)	7.5(6–11)	0.22
Previous antibiotic therapy	17(41%)	61(95%)	6(60%)	< 0.001
Aminoglycoside	5(12%)	18(28%)	2(20%)	0.13
Fluoroquinolone	3(7%)	20(31%)	2(20%)	0.009
Macrolide	3(7%)	13(20%)	2(20%)	0.15
Beta-lactam/Beta-lactamase inhibitor	4(10%)	27(42%)	4(40%)	0.001
Cephalosporin	5(12%)	10(16%)	0(0%)	0.49
Carbapenem	1(2%)	22((34%)	0(0%)	< 0.001
Tigecycline	0(0%)	4(6%)	0(0%)	0.29
Clindamycin	0(0%)	6(9%)	0(0%)	0.12
Vancomycin or teicoplanin	0(0%)	23(36%)	1(10%)	< 0.001
Metronidazole	0(0%)	7(11%)	0(0%)	0.06
Duration of prior antibiotics used (days)	0(0–6)	7(6–8)	4(0–5)	< 0.001
Health-care-associated infection		61(95%)	2(20%)	< 0.001

Note: Values are in median (IQR), number, number (%)

Patients with MDR-GNB in comparison to non-MDR-GNB were found to have high incidence of previous antibiotic therapy (95%, 61/64 versus 60%, 6/10; p = < 0.001) and HCAI (95%, 61/64 versus 20%, 2/10; *p* = < 0.001).

With respect to the clinical outcome, in-hospital-mortality among patients in the MDR group (38%, 24/64) was significantly higher than those in the non-MDR group (20%, 2/10) and uninfected group (10%, 4/41) (*p* = 0.007) as depicted in Table 4. However, no difference was detected when MDR-GNB group was compared to non-MDR-GNB group (*p* = 0.47).

**Table 4:** Clinical outcome of patients

Outcome	Uninfected patients *n* = 41	Patients with MDR-GNB *n* = 64	Patients with non-MDR-GNB *n* = 10	*p*-value
In-hospital-mortality	4(10%)	24 (38%)	2 (20%)	0.007
Discharged	37 (90%)	40 (62%)	8 (80%)	0.007
ICU stay	9(5–12)	13(8–18)	9(7–12)	0.43
Hospital stay	11(8–17)	14(10–21)	9(7–15)	0.93

Note: Values are in median (IQR), number, number (%)

The findings of univariate and multivariate logistic regression for variables associated with in-hospital- mortality are described in Table 5.

**Table 5:** Univariate and Multivariate logistic regression for variables associated with hospital mortality

Variables	Univariate analysis		Multivariate analysis	
	Odds ratio (95% CI)	*p* value	Odds ratio (95% CI)	*p* value
MDR GNB^a^	5.46(1.72–17.26)	0.004	4.71(1.42–15.54)	0.01
Non- MDR GNB^a^	2.37(0.36–15.26)	0.36	2.60(0.38–17.83)	0.32
Age	1.00(0.98–1.03)	0.50	1.001(0.96–1.03)	0.95
Male	0.70(0.29–1.66)	0.42	0.59(0.23–1.53)	0.28
CCI Score	1.15(0.84–1.56)	0.36	1.05(0.63–1.72)	0.84
APACHE 24 h	1.07(1.00–1.16)	0.04	1.04(0.91–1.20)	0.48
APACHE 48 h	1.09(1.00–1.17)	0.02	0.38(0.89–1.17)	0.72

Note: ^a^In reference to patients without infection

After adjustment for independent risk factors, compared to uninfected patients, the odds ratio (CI) for in-hospital-mortality in MDR-GNB group was (4.7[1.4–15.5], *p* = 0.01), while in patients with non-MDR-GNB it was (2.60 [0.38–17.8], *p* = 0.32).

## Discussion

The increasing incidence of MDR-GNB infections reported from the different ICU’s in Nepal is of great concern [6, 7]. However, most prior work from Nepal has been focused on their incidence and the common mechanism of drug resistance [6, 7]. To our knowledge, this is the first study from Nepal that highlights the association between MDR-GNB infections and various clinical outcomes in ICU admitted patients.

The present study found that MDR-GNB infections was not uncommon in ICU and it accounted for 47 MDR-GNB cases per 100 ICU admission. Despite significant advances in ICU in current years, the incidence of MDR-GNB HCAI remains higher in the ICU compared with other hospital units [20]. In our study, 95% cases of MDR-GNB were associated with HCAI. Similar findings were reported by other recent studies from Nepal which were done by Parajuli et al., Bhandari et al. and khanal et al. which reported 96% [6], 79% [21] and 69% [7] of GNB causing HCAI from ICU were MDR. Rampant antibiotic use, increased prevalence of drug resistance and nonadherence to infection control strategies are the emerging problems in Nepalese ICU’s predisposing for the emergence and spread of HCAI [6]. Likewise, in a study from India, 58% MDR-GNB were isolated from the ICUs specimens from the total received specimens [22]. Another study from India on epidemiology of MDR-GNB isolated from ventilator-associated pneumonia in ICU patients found 88% of total isolates to be GNB, among which 72% were MDR [23]. A systematic review of the burden of MDR HCAI among ICU patients in Southeast Asia showed substantially higher incidence of MDR *Acinetobacter baumannii* (58%) than reported from other parts of globe [24]. These scenario shows high prevalence of MDR-GNB infections in ICUs of Asia including Nepal. The present study showed high frequency of bacterial isolates producing beta-lactamases (MBL 59%, ESBL 24%). Current studies from Nepal also have reported high incidence of ESBL (43% [6], 40% [21], 25% [7]) and MBL (65% [21], 50% [6], 37% [7]) from ICU. Prevalence of ESBL and carbapenemases producing GNB from ICU was 22.7% and 9.6% respectively in a recent study from India [25]. Studies from the west also have shown an increasing trend of ESBL with ICU GNB isolates [20]. Sader and colleagues reported on the prevalence and trends of MDR-GNB occurring in the ICU of the hospitals in the United States and Europe from January 2009 to December 2011 [20]. Over the 3-year study period, rates of ESBL-producing strains of *Escherichia coli* and *Klebsiella spp* from the ICU increased from 11.9 to 17.4% and 27.5–41.8% respectively from 2009 to 2011 [20]. Alike, a SENTRY study also reported that GNB resistance to imipenem increased from 34.5% in 2006 to 59.8% in 2009 across the world [26]. This globally increasing trend of carbapenemase resistance in the ICUs poses a significant concern since it limits the range of therapeutic alternative forcing the clinicians to use agents like colistin which is expensive and associated with significant toxicity [8]. The reports of infections caused by MDR non-fermentative gram-negative bacteria and enterobacteriaceae are increasingly documented from the Nepalese ICU. In this study, 93% of *Acinetobacter spp,* 86% of *Klebsiella pneumoniae*, 84% of *Pseudomonas spp* and 64% of *Escherichia coli* were MDR and a similar result was also reported from Nepal [7]. Excessive use of broad spectrum antibiotics as observed in this study along with inadherence to infection control measures are the main causes for this terrifying rates of MDR infections in our ICU.

In the present study, multivariate analysis showed strong association between MDR-GNB patients and in-hospital-mortality even after adjusting all the confounding factors (Odds ratio: 4.7, *p*-0.01). Ben-David D et al. [11], in a retrospective study on the outcome of carbapenem-resistant *Klebsiella pneumoniae,* (CRKP) BSI, also found mortality to be significantly higher among patients with CRKP compared with those with susceptible *K. pneumoniae* BSI (48% vs.17%). A study by Cosgrove et al. [10] on the impact of the emergence of resistance to third-generation cephalosporins in *Enterobacter spp* on patient outcomes also found a significant increase in mortality (Relative risk, 5.02). This may possibly due to that appropriate antibiotic therapy will be started later for MDR-GNB infections in compared to infections caused by antibiotic-sensitive bacteria. In contrary to our findings some of the earlier studies did not find significant associations between MDR-GNB and mortality [12, 13]. However, variation in the clinical virulence of the varieties of GNB prevalent in different geographical areas may be the reasons for these conflicting results. Further, the patients infected by MDR-GNB, compared with those with non-MDR-GNB isolates, had a longer average stay in ICU and hospital, however, it did not reached the statistically significant level. As a consequence of prolonged hospitalization, MDR-GNB patients may have the economic impact due to increase in financial burden.

This study had certain limitations, including small sample size and lack of data on inappropriate empiric antibiotic therapy that could possibly influence in-hospital-mortality. Also, genotypic screening for resistance genes could not be performed due to the limited resources.

## Conclusion

The present study revealed a high incidence of MDR-GNB infections in ICU. HCAI and in-hospital-mortality were significantly associated with MDR-GNB infection. Likewise, MDR-GNB patients needed prolong ICU and hospital stay, however, it was statistically insignificant. Our study highlights the alarming need of multidisciplinary efforts to address the situation and recommends the implementation of antimicrobial stewardship, continuous surveillance, strict adherence to hand hygiene and contact precautions and regular environmental cleaning to contain the development and spread of antimicrobial resistance among the local isolates.

## Abbreviations

APACHE II: Acute physiology and chronic health evaluation II
BPKIHS: B.P Koirala Institute of Health Sciences
BSI: Blood stream infection
CCI: Charlson comorbidity index
CDC: Centers for Disease Control and Prevention (CDC)
CLSI: Clinical Laboratory Standard Institute
ECDC: European Centre for Disease Prevention and Control
ESBL: Extended-spectrum beta-lactamases
HCAI: Healthcare-associated infections
ICU: Intensive care unit
IRC: Institutional review committee
LOS: Length of stay
LRTI: Lower respiratory tract infection
MDRGNB: Multi-drug resistant gram-negative bacilli
MHA: Mueller Hinton agar (MHA)
MHT: Modified hodge test
SSI: Surgical site infection
UTI: Urinary tract infection
XDR: Extensively drug resistant

## References

[1] Vincent JL, Sakr Y, Sprung CL (2006). Sepsis in European intensive care units: results of the SOAP study. Crit Care Med.

[2] Vincent JL, Rello J, Marshall J (2009). International study of the prevalence and outcomes of infection in intensive care units. JAMA.

[3] Cohen J (2013). Confronting the threat of multidrug-resistant gram-negative bacteria in critically ill patients. J Antimicrob Chemother.

[4] Chaudhry D, Prajapat B (2017). Intensive care unit bugs in India: How do they differ from the Western world?. J Assoc Chest Physicians.

[5] Mendes Rodrigo E., Mendoza Myrna, Banga Singh Kirnpal K., Castanheira Mariana, Bell Jan M., Turnidge John D., Lin Stephen S. F., Jones Ronald N. (2013). Regional Resistance Surveillance Program Results for 12 Asia-Pacific Nations (2011). Antimicrobial Agents and Chemotherapy.

[6] Parajuli NP, Acharya SP, Mishra SK (2017). High burden of antimicrobial resistance among gram-negative bacteria causing healthcare associated infections in a critical care unit of Nepal. Antimicrob Resist Infect Control.

[7] Khanal Santosh, Joshi Dev Raj, Bhatta Dwij Raj, Devkota Upendra, Pokhrel Bharat Mani (2013). β-Lactamase-Producing Multidrug-Resistant Bacterial Pathogens from Tracheal Aspirates of Intensive Care Unit Patients at National Institute of Neurological and Allied Sciences, Nepal. ISRN Microbiology.

[8] Boucher HW, Talbot GH, Bradley JS (2009). Bad bugs, no drugs: no ESKAPE! An update from the Infectious Diseases Society of America. Clin Infect Dis.

[9] Falagas ME, Bliziotis IA, Kasiakou SK (2005). Outcome of infections due to pan-drug resistant (PDR) gram-negative bacteria. BMC Infect Dis.

[10] Cosgrove SE (2006). The relationship between antimicrobial resistance and patient outcomes: mortality, length of hospital stay, and health care costs. Clin Infect Dis.

[11] Ben-David D, Kordevani R, Keller N (2012). Outcome of carbapenem resistant Klebsiella pneumoniae bloodstream infections. Clin Microbiol Infect.

[12] Blot S, Vandewoude K, De Bacquer D (2002). Nosocomial bacteremia caused by antibiotic-resistant gram-negative bacteria in critically ill patients: clinical outcome and length of hospitalization. Clin Infect Dis.

[13] Menashe G, Borer A, Yagupsky P (2001). Clinical significance and impact on mortality of ESBL-producing gram-negative isolates in nosocomial bacteremia. Scand J Infect Dis.

[14] Washington CW, Stephen DA, William MJ (2006). Koneman’s color atlas and text book of diagnostic microbiology.

[15] Clinical and Laboratory Standards Institute. Performance standards for antimicrobial susceptibility testing; 27th ed. CLSI supplement. *CLSI Document M100-S27*. Wayne, PA: Clinical and Laboratory Standards Institute; 2017.

[16] Yong D, Lee K, Yum JH (2002). Imipenem-EDTA disk method for differentiation of metallo-beta-lactamase-producing clinical isolates of pseudomonas spp. and Acinetobacter spp. J Clin Microbiol.

[17] Magiorakos AP, Srinivasan A, Carey RB (2012). Multidrug-resistant, extensively drugresistant and pandrug-resistant bacteria: an international expert proposal for interim standard definitions for acquired resistance. Clin Microbiol Infect.

[18] Knaus WA, Draper EA, Wagner DP (1985). APACHE II: a severity of disease classification system. Crit Care Med.

[19] Charlson ME, Pompei P, Ales KL (1987). A new method of classifying prognostic comorbidity in longitudinal studies: development and validation. J Chronic Dis.

[20] Sader HS, Farrell DJ, Flamm RK, Jones RN (2014). Antimicrobial susceptibility of gram-negative organisms isolated from patients hospitalized in intensive care units in United States and European hospitals (2009-2011). Diagn Microbiol Infect Dis.

[21] Bhandari P, Thapa G, Pokhrel BM (2015). Nosocomial Isolates and Their Drug Resistant Pattern in ICU Patients at National Institute of Neurological and Allied Sciences, Nepal. Int J Microbiol.

[22] Subhedar V, Jain SK (2016). Gram negative super bugs: a new generation of ICU infections, an emerging challenge for health care settings. Am J Microbiol Res.

[23] Gupta R, Malik A, Rizvi M (2017). Epidemiology of multidrug-resistant gram-negative pathogens isolated from ventilator-associated pneumonia in ICU patients. J Glob Antimicrob Resist.

[24] Teerawattanapong N, Panich P, Kulpokin D (2018). A systematic review of the burden of multidrug-resistant healthcare-associated infections among intensive care unit patients in Southeast Asia: the rise of multidrug-resistant Acinetobacter baumannii. Infect Control Hosp Epidemiol.

[25] Arora A, Jain C, Saxena S, Kaur R (2011). Profile of drug resistant gram negative bacteria from ICU at a tertiary Care Center of India. Asian J Med Health.

[26] Gales AC, Jones RN, Sader HS (2011). Contemporary activity of colistin and polymyxin B against a worldwide collection of gram-negative pathogens: results from the SENTRY antimicrobial surveillance program (2006-09). J Antimicrob Chemother.

